# Screening migrants for tuberculosis - a missed opportunity for improving knowledge and attitudes in high-risk groups: A cross-sectional study of Swedish-language students in Umeå, Sweden

**DOI:** 10.1186/1471-2458-10-349

**Published:** 2010-06-17

**Authors:** Faustine KK Nkulu, Anna-Karin Hurtig, Clas Ahlm, Ingela Krantz

**Affiliations:** 1 Department of Public Health and Clinical Medicine, Epidemiology and Global Health, Umeå University, Umeå, SE-90 185, Sweden; 2 Department of Clinical Microbiology, Division of Infectious Diseases, Umeå University, Umeå, SE-901 85, Sweden; 3 Skaraborg Institute for Research and Development, Skövde, SE-541 30, Sweden

## Abstract

**Background:**

Migrants from countries with a high-burden of tuberculosis (TB) are at a particular risk of contracting and developing the disease. In Sweden, new immigrants are routinely offered screening for the disease, yet very little is known about their beliefs about the disease which may affect healthcare-seeking behaviours. In this study we assessed recent immigrant students' knowledge of, and attitudes towards TB, and their relationship with the screening process.

**Methods:**

Data were collected over a one-year period through a survey questionnaire completed by 268 immigrants consecutively registered at two Swedish-language schools in Umeå, Sweden. Participants originated from 133 different countries and their ages varied between 16-63 years. Descriptive and multivariate logistic regression analyses were then performed.

**Results:**

Though most of them (72%) were screened, knowledge was in general poor with several misconceptions. The average knowledge score was 2.7 ± 1.3 (SD), (maximum = 8). Only 40 (15 %) of the 268 respondents answered at least half of the 51 knowledge items correctly. The average attitude score was 5.1 ± 3.3 (SD) (maximum = 12) which meant that most respondents held negative attitudes towards TB and diseased persons. Up to 67% lacked knowledge about sources of information while 71% requested information in their vernacular. Knowledge level was positively associated with having more than 12 years of education and being informed about TB before moving to Sweden. Attitude was positively associated with years of education and having heard about the Swedish Communicable Disease Act, but was negatively associated with being from the Middle East. Neither knowledge nor attitude were affected by health screening or exposure to TB information after immigration to Sweden.

**Conclusions:**

Though the majority had contact with Swedish health professionals through the screening process, knowledge about tuberculosis among these immigrants was low with several misconceptions and negative attitudes. Information may currently be inaccessible to most of these immigrants due to the language barrier and unfamiliarity with the Swedish healthcare system. If TB education was included as a component of screening programmes, ensuring that it was tailored to educational background, addressed misconceptions and access problems, it could well help improve TB control in these communities.

## Background

Tuberculosis (TB) is one of the most serious health problems globally. About one third of the global population is estimated to be infected with *Mycobacterium tuberculosis *which kills an estimated two million of the approximately eight million people who develop the disease each year [1,2]. The World Health Organisation (WHO) declared TB a global health emergency in 1993 following dramatic changes in the magnitude of the problem. However, the number of new cases continues to increase and without a coordinated control effort, TB will infect an estimated one billion more people by 2020, killing 36 million [1,2]. This resurgence is due to several interrelated factors, including the HIV epidemic, ineffective control-programmes, population growth, and the increasing geographical movement of people either already with the disease, or those at risk of contracting and developing TB [1-4].

The growing international migration is thought to be the greatest factor contributing to the rise in cases and changes observed in the epidemiology of TB in the developed world. Studies conducted throughout the 1990s and recently in North America, Western Europe and Australia attribute a large proportion of cases to foreign-born residents [3-6]; following this pattern, immigrants to Sweden constitute an increasingly high share of the TB cases reported since 1992 [7-10]. The proportion of foreign-born cases, which in 1989 was 202 (34%) of 595 reported cases, had more than doubled and reached 83% of the 554 cases reported in 2008 [7-10].

Regardless of whether infection occurs as a reactivation of an old infection, a re-infection, a new infection in the host country, or through frequent visits to the home country, the risk of TB among immigrants from highly endemic areas remains high many years after immigration [7-12]. For instance, 106 (23%) of the 460 foreign-born cases reported in 2008 had resided in Sweden for more than ten years [10]. Likewise, most of the cases (82/102) in the five outbreaks of an identical Isoniazid resistant strain of *M. tuberculosis*, that occurred in Sweden between 1996 and 2005, were young immigrants from Sub-Saharan Africa who had migrated to Sweden more than five years earlier [7].

The Swedish Communicable Disease Act identifies TB as a public health threat, thus it is a notifiable disease. Screening of foreign-born persons from high-burden countries (all countries in the world except countries in North America, Western Europe, Australia and New Zealand) is initiated with a tuberculin skin test, linked to the first health screening following their entrance into the country [13]. The main purpose of the screening is to discover asymptomatic or symptomatic patients who are eligible for preventive or curative treatment and to stop further transmission. In addition to the screening of high-risk groups, TB is controlled in Sweden by contact tracing, which investigates whether TB has been spread through previous contacts that TB patients may have had, while vaccination of at-risk children (those born in high-burden countries or to parents from such countries) is used to reduce the more severe consequences of TB [13]. Screening of some subgroups of foreign-born persons for TB is widely used in many developed countries to meet the challenge of migrant-associated TB [3,5,11-14]. Adequate knowledge of TB is important for healthcare seeking, adherence to treatment and preventive measures [15-17]. In Sweden, despite the wide recognition of TB as a major health issue among some subgroups of migrants, very little is known about their knowledge of, beliefs about, and attitudes towards TB, all of which may be determinants for healthcare-seeking behaviours.

In this study we assessed migrant students' knowledge of, and attitudes towards TB, and their relationship with the screening process.

## Methods

### Study-design and setting

A cross-sectional study was carried out between October 2007 and September 2008 in two purposively-selected municipal language schools for immigrants in the city of Umeå, northern Sweden. In December 2007, Umeå was home to about 111,571 inhabitants including 9,577 immigrants, of these, 35% originated from Asia, 26% from other Nordic Countries, 12 % from other European Union states, 12% from Africa, 7% from other European countries and 4% from South America. North America, Australia, New Zealand and the former Soviet Union made up together about 4%. Umeå municipality offers Swedish language training at no cost to all recent legal immigrants at four different locations depending on age and previous education; the first of these is located in one of the municipal primary schools, and is intended for children aged 6-15; the second location was one of our study settings where young immigrants (16-20) attended the Individual Programme's Introductory Course (IVIK: *Individuellt Program Introduktionskurs*) in one of the municipal high-school premises. The third location is the SFI (*Svenska För Invandare, or Swedish for Immigrants*) adult (18 and > ) school, which is the largest and was also the second location for our study. Finally, those who hold a high school degree from their home country, have good knowledge of English, and intend to pursue their university studies (25-30 students per academic year) can learn Swedish at Umeå University. The IVIK and SFI schools were convenient choices for the study because these schools enroll students throughout the year, had the highest number of attendees and also because there was no TB screening center in Umeå. Immigrants are screened at the nearest health center depending on where they live. Those who test positive (PPD ≥ 10 mm) on the tuberculin test are then referred to the Lung and Allergy Clinic at the University Hospital for further investigation and treatment.

### Study participants

Eligible study-participants were newly-arrived immigrants aged 16 or over and enrolled at the schools under the study period. The two inclusion criteria were, first, being registered as a full time student at one of the selected two schools and, secondly, speaking a language spoken by at least three other students. According to the school register records, 102 youth and 490 adult immigrants were formally registered as students in the two schools under the study period. However, after excluding drop outs, those on sick or maternal leave, and part-time students, no more than 378 attendees remained, of which 276 were regular attendees at SFI classes (N = 276) and 102 youths enrolled on the Individual Programme's Introductory Course at one of the municipality high schools (N = 102) under the study period. Only 22 of the 55 contacted parents/guardians of the under 18s, however, sent back a signed informed consent form, and three later withdrew their children from the study; thus, we could not include 33(32%) of the 102 eligible young students without parental consent. In addition, on the days of the survey, 23 (23%) of the102 child students and 10/276 (4%) of the adult students could not participate due to unavailability of interpreters. Participation was purely voluntary and some students might have chosen not to participate, but no specific details of non-respondents could be obtained since teachers unfortunately failed to provide us with attendance lists for data collection days. We could, however, estimate the response rates at 268 (71%) of the 378 officially registered. The total analysis is thus based on 268 (96%) of the 280 individuals who completed the questionnaire. We adopted Statistics Sweden's definition of an immigrant which defines a migrant as foreign-born, legally admitted and expected to stay at least 12 months in Sweden [18].

### Data collection instrument and procedure

A pretested questionnaire developed by the first author for the purpose of this study based on relevant literature and information material was anonymously administered to all attendees [19,20]. The original questionnaire included 80 knowledge and 15 attitudinal items with which they could agree or disagree. Each knowledge item was placed in one of the following eight topics: causes, mode of transmission, high-risk groups, symptoms, diagnosis, latent infection, and treatment & prevention. Attitudinal items were grouped under two topics: attitude towards the disease (which included statements about how participants might react if they had TB), and attitude towards diseased persons (where the statements were about how participants might react if they had a close relative or friend with TB). Also added were a set of socio-demographic questions and a series of questions related to perceived risk of contracting the disease, seriousness of the disease and TB information made available in Sweden. The questionnaire was translated and printed in Swedish, English and French.

The interviews were conducted on six separate occasions (two at the youth school and four at the adult school) to allow for the inclusion of newcomers. Interpreters and international master's students in public health were hired to overcome language and literacy barriers. They were handed the questionnaire a few days beforehand for translation, and on the survey day the questionnaire was read aloud by the interviewer in the respondent's vernacular for respondents who then checked the appropriate option. Face-to-face interviews were used for 17 respondents who were unable to fill in the questionnaire by themselves. Of the 268 respondents who participated, 6 were interviewed in Amharic, 71 in Arabic, 38 in Badinani, 9 in Chinese, 3 in Dari, 28 in English, 22 in French, 3 in Japanese, 13 in Persian, 23 in Somali, 4 in Sorani, 13 in Spanish, 8 in Swahili, 23 in Thai, and 4 in Tigrinya.

### Data recording and analysis

The response options were "Yes", "No", and "Don't Know" for knowledge items and "Agree", "Disagree", and "No Opinion" for attitudinal items. Descriptive statistics were initially performed to summarize the data; thereafter two new variables, "TB knowledge score" and "TB attitude score", were created for the purpose of the logistic regression analyses.

### TB knowledge score

After testing correlation between items for each knowledge topic, 51 knowledge items were retained as valid and included in the calculation of knowledge score as follows: for each individual item, correct answers were re-coded into "1" and "incorrect" or "do not know" answers into "0". A single, summary score for each knowledge topic was obtained by summing the number of correct responses and dividing the total by the number of items in the topic. An aggregate measure was then computed, summing the values of each topic, yielding a knowledge score for each participant. The theoretical range of knowledge was 0-8, with a higher score indicating a higher level of knowledge. The average knowledge-scores for the group were obtained by summing each participant's score and dividing it by the total number of participants. For the purpose of the analysis, the cumulative scores were pragmatically further dichotomized into 'low' and 'high' levels of knowledge. Respondents with a total score equal or above 4 were coded as having 'high' or 'good' knowledge and those below 4 as having 'low' or 'bad' knowledge.

### TB attitude score

After testing correlation, only 12 attitudinal items were retained as valid and included in the calculation of attitude score. For each individual item, agreeing with a rational statement, or disagreeing with an irrational one, was re-coded into"1". Agreeing with an irrational statement, or disagreeing with a rational one, as well as "No Opinion" answers were re-coded into "0". Then, the same process as for TB knowledge was repeated to follow a similar pattern, the only difference being that due to the low number of items, the attitude score was obtained by summing the values of each attitudinal item. Attitude scores ranged between 0-12, with a higher score indicating a more positive/tolerant attitude. Then the same process as above was repeated to obtain the average attitude score and dichotomize scores into positive and negative attitudes. Respondents with a total score above 6 were coded as having a positive/tolerant attitude, compared to those with a score equal to or below 6 being coded as having a negative/stigmatising attitude.

Univariate and multivariate logistic regression analyses with 95% confidence intervals were performed using the Statistical Package of Social Science (SPSS Inc, Chicago, IL) for Windows version 15.0. A *p*-value of < 0.05 was considered statistically significant.

### Ethical consideration

The research proposal was approved by the Doctoral Education Board (Faculty of Medicine) of Umeå University. Permission to carry out the study was obtained from the principals of the two schools where the survey was conducted. Parents/guardians were given the opportunity to withdraw their children from the study if they were opposed. Prior to administration of the questionnaire, an introductory letter attached to the questionnaire was read to the participants in their vernacular, clarifying both the purpose and the voluntary nature of the study. The letter also summarized issues of confidentiality, anonymity, informed consent, and the importance of honesty in the responses. Participants were also given the opportunity to withdraw from the study at anytime.

## Results

### Socio-demographic characteristics

Table 1 presents the demographic characteristics of respondents by sex. There were 147 male and 121 female attendees aged between 16-63 years (mean age 30). Respondents originated from 133 different countries and spoke 25 different languages or dialects. Most respondents (80%) had been residing in Sweden for less than four years, only three respondents had moved to Sweden more than ten years ago.

**Table 1:** Respondents' Socio-Demographic Characteristics by Sex (N = 268)

Characteristics		Males = 147	Females = 121	Total = 268
		N (%)	N (%)	N (%)
**Age**	16-24	56 (38.1.)	25 (20.7)	81 (30.2)
	25-35	57 (38.8)	48 (39.7)	105 (39.2)
	35-44	20 (13.6)	32 (26.4)	52 (19.4)
	45+	9 (6.1)	1 (9.1)	20 (7.5)
	N/A	5 (3.4)	5 (4.1)	10 (3.7)

**Schooling**	0-6 years	30 (20.4 )	24 (19.8)	54 (20.1)
	7-12 years	62 (42.2)	39 (32.2)	101(37.7)
	13+ years	45 (30.6)	50 (41.3)	95 (35.5)
	N/A	10 (6.8)	8 (6.6)	18(6.7)

**Marital status**	Single	71 (48.3)	18 (14.9)	89 (33.2)
	Married or living as couples	53 (36.1)	81 (66.9)	134 (50)
	Divorced/Separated/Widowed	19 (12.9)	20(16.5)	39 (14.6)
	N/A	4 (2.7)	2 (1.7)	6 (2.2)

**Immigration/legal status**	Refugees	98 (66.6)	42 (34.7)	140 (52.2)
	Immigrants (Students +Workers)	6 (4.1)	5 (4.1)	11 (4.1)
	Dependent to immigrants/refugees	15 (10.2)	38 (31.4)	53(19.8)
	Dependent to Swedes	12 (8.2)	32 (26.5)	44 (16.4)
	N/A	16 (10.9)	4 (3.3)	20 (7.5)

**Geographic origin**	Middle East	94 (63.9)	39 (32.2)	133(49.6)
	Sub-Saharan Africa	37 (25.2)	31 (25.6)	68 (25.4)
	Asia	6 (4.1)	34 (28.1)	40 (14.9)
	Latin America	4 (2.7)	9 (7.5)	13 (4.9)
	Low-risk countries	6 (4.1)	8 (6.6)	14 (5.2)

**Religion**	Muslim	104 (70.7)	46 (38)	150 (56)
	Christian	32 (21.8)	35 (28.9)	67 (25)
	Atheist	5 (3.4)	15 (12.4)	20 (7.5)
	Other	4 (2.7)	25 (20.7)	29 (10.8)
	N/A	2 (1.4)	0	2 (0.7)

**Health check up**	Yes	115 (78.2)	78 (64.5)	193 (72)
	No	32 (21.8)	43 (35.5)	75 (28)

### TB knowledge

**Figure 1:**
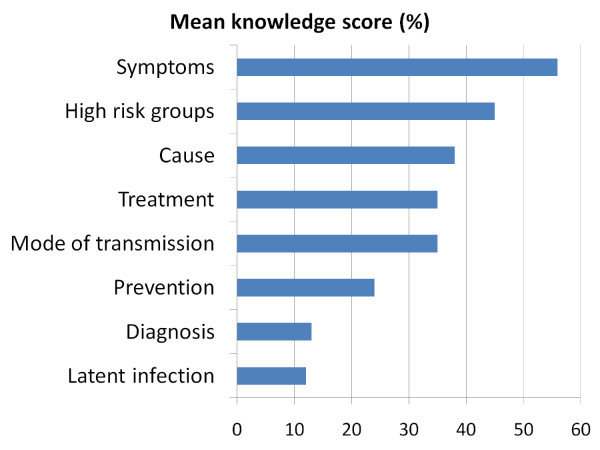
**Proportion of maximal mean knowledge score**. Mean knowledge scores by topic as percentages of maximal scores

Knowledge was in general poor (Table 2) with several misconceptions (Table 3). The average knowledge score for the group was 2.7 ± 1.3 (SD), maximum = 8. Only 40 (15 %) of the 268 respondents answered at least half of the 51 knowledge items correctly. On an overall one-point scale, respondents scored on average rather well about symptoms, treatment and which groups were at high risk of TB infection/disease. However, the students showed poor knowledge about cause, mode of transmission, prevention, diagnosis and latent infection (Figure 1). About half of the respondents described TB as a viral disease (45%) that could be transmitted through blood transfusion (49%), sharing cigarettes (52%) or kitchen utensils (53%). More than half of respondents believed that everyone infected with TB becomes sick (65%) and infectious (68%). In addition, although, nearly half (45%) of respondents correctly agreed that a TB treatment course lasts between six to nine months, some others inaccurately agreed that it takes less than six months (13%) or more than a year (28%)(Table 3). The association between socio-demographic characteristics and knowledge level was estimated by crude odds ratio, and then adjusted to control for the effect of prior knowledge and geographic origin (religion was not included in the analysis because it was correlated with geographic origin) (Table 4). Only previous education (*P*_0-6 _= 0.044, *P*_7-12 _= 0.019) and having heard about TB before migration were significantly (*P *= 0.025) associated with knowledge level.

**Table 2:** Knowledge about TB among Immigrant Students in Umeå Swedish-Language Schools (N = 268)

Items accurately identified by most respondents as correct	Number of correct answers (%)
**TB is caused by a bacterium**	152 (56.7)
**TB can spread to other people if a diseased person:**	
Coughs	203 (75.8)
Shares a room with them	140 (52.2)
Spits	136 (50.8)
Sneezes	135 (50.4)
**TB is not transmitted through handshakes**	143 (53.4)
**People at increased risk include:**	
Those in contact with sick people	192 (71.6)
Smokers	159 (59.3)
Prisoners	147 (54.9)
Substance abusers	145 (54.1)
HIV infected	144 (53.7)
Homeless	138 (51.5)
**Symptoms related to TB:**	
Coughing up blood	202 (75.4)
Chest pain	166 (61.9)
Feeling tired all the time	160 (59.7)
Fever	152 (56.7)
Unexplained weight loss	148 (55.2)
**To get rid of TB, patients must continue treatment even if they feel better after two-three weeks**	196 (73.1)
**To prevent the spread of TB, all patients should be asked to:**	
spit in a tissue and avoid coughing on others	200 (74.6)
sleep in a bedroom away from other family members	186 (69.4)
cover their noses when coughing or sneezing	183 (68.3)

**Table 3:** Common Misconceptions about TB among Immigrant Students in Umeå Swedish-Language Schools (N = 268).

Items inaccurately identified as correct by respondents	Number of "Yes" answers* (%)
**TB is caused by a virus**	120 (44.8)
**TB is transmitted through:**	
Sharing dishes/kitchen utensils	143 (53.4)
Sharing cigarettes	140 (52.2)
Blood transfusion	132 (49.3)
Breastfeeding	121(45.2)
Hugs	65 (24.3)
**TB cannot spread to other people if a diseased person:**	
Laughs	109 (40.7)
Sings	109 (40.7)
Speaks	97 (36.2)
**Persons not at increased risk of TB:**	
Diabetic patients	87 (32.5)
Health care workers	77 (28.7)
Persons with Cancer	76 (28.4)
**A short-lived cough (one-two weeks) is a symptom of TB disease**	144 (53.7)
**A positive skin test without symptoms suggests:**	
A need for curative treatment	168 (62.7)
Infectious TB	138 (51.5)
Active TB disease	123 (45.9)
No previous vaccination	69 (25.8)
**TB patients remain infectious two to three weeks after beginning treatment**	161 (60.1)
**Everyone infected with TB bacteria:**	
Can spread TB to others	182 (67.9)
Become sick	175 (65.3)
Have symptoms	143 (53.4)
**Key methods to prevent the spread of TB:**	
Forbid sick people to eat from the same plate as other family members	177 (66.1)
Stay away from everyone who coughs	159 (59.3)

*Don't know responses are not included

**Table 4:** Factors Associated with Students' Level of TB knowledge

Variable	Proportion with good knowledge N (%)	Crude Odds ratio	95% CI	Adjusted Odds ratio	95% CI
**Sex**					
Men	16 (10.9)	0.494	0.249 0.979	0.723	0.299 1.749
Women	24 (19.8)	1.0		1.0	

**Age**					
16-24	9 (11.1)	0.5	0.137 1.828	0.271	0.058 1.269
25-34	19 (18.1)	0.884	0.265 2.943	0.754	0.199 2.862
35-44	6 (11.5)	0.522	0.130 2.089	0.384	0.074 1.996
45+	4 (20)	1.0		1.0	

**Schooling**					
					
0-6 Years	3 (5.5)	0.191	0.054 0.673*	0.231	0.056 0.961*
7-12 Years	11 (10.9)	0.406	0.185 0.891*	0.286	0.101 0.815*
13+ Years(ref)	22 (23.2)	1.0		1.0	

**Geographic origin**					
Middle East	9 (6.8)	0.131	0.036 0.473*	0.267	0.062 1.150
Asia	6 (15.0)	0.318	0.079 1.283	0.291	0.059 1.435
Latin America	1 (7.7)	0.15	0.015 1.518	0.246	0.018 3.414
Sub- Saharan Africa	19 (27.9)	0.698	0.207 2.352	2.315	0.473 11.378
Low incidence countries	5 (35.7)	1.0		1.0	

**Health check up**					
Yes	26 (13.5)	0.678	0.333 1.384	0.681	0.284 1.629
No	14 (18.5)	1.0		1.0	

**Have heard about TB before migration**					
Yes	29 (20.3)	2.636	1.257 5.531*	3.025	1.145 7.990*
No	11 (8.8)	1.0		1.0	

**Received TB information in Sweden**					
Yes	13 (16.9)	1.234	0.599 2.540	0.658	0.255 1.695
No	27 (14.1)	1.0		1.0	

**Heard about the Communicable Disease Act**					
Yes	10 (16.1)	1.128	0.517 2.460	1.055	0.386 2.882
No	30 (14.6)	1.0		1.0	

*P < 0.05 (Univariate and multivariate logistic regression with TB knowledge level as dependent variable)

### Attitudes towards TB and diseased persons

Negative attitudes towards the disease and diseased persons were common. Most respondents would rather isolate themselves if they had TB. They also reported a likely fear of spreading the disease and said they would be worried about others' reactions if they found out they had contracted TB. Only approximately 30% of respondents did not express such a fear. More respondents would rather disclose their illness only to family members (60%) than friends (48%); the remainder would be embarrassed to do so. Some respondents expressed an undue fear of seeking medical attention or to be deported because of TB, but the majority were without fear or ambivalent.

When asked about how they would react if they had a relative or close friend suffering from TB, 'avoiding her/him' was the most common attitude among respondents. Slightly more than half of respondents indicated that they would not share food with them (51%). Merely 40% of respondents pragmatically affirmed that they would give them a hug (37%), feel comfortable to travel in the same vehicle (43%) or be in the same classroom (40%). The overall average attitude score was 5.1 ± 3.3 (SD) (maximum = 12) meaning that most respondents had a negative attitude towards TB and diseased persons. The association between socio-demographic characteristics and attitude score was estimated by crude odds ratio and then adjusted to control for the effect of sex, prior knowledge, the extent of knowledge, and geographic origin (Table 5). Attitude was positively associated with more than 12 years of education (P = 0.008) as was having heard about the Communicable Disease Act (*P *= 0.032). Attitude was negatively associated with being from the Middle East (*P *= 0.002).

**Table 5:** Factors Associated with Students' Attitudes towards TB and Diseased People

Variable	Proportion with positive attitude	Crude Odds ratio	95% CI	Adjusted Odds ratio	95% CI
**Sex**					
Men	56 (38.1)	0.480	0.294 0.783*	0.879	0.414 1.870
Women	68 (56.2)	1.0		1.0	

**Age**					
16-24	36 (44.4)	0.978	0.366 2.616	0.507	0.125 2.054
25-34	51 (48.6)	1.154	0.442 3.016	0.911	0.253 3.282
35-44	24 (46.2)	1.048	0.372 2.952	0.727	0.177 2.982
45+	9 (45.0)	1.0		1.0	

**Education group/level (years in school)**					
0-6 Years	23 (41.8)	0.57	0.291 1.115	0.405	0.153 1.074
7-12 Years	38 (37.6)	0.478	0.270 0.846*	0.308	0.129 0.734*
13+ Years(ref)	53 (55.8)	1.0		1.0	

**Geographic origin**					
Middle East	23 (17.3)	0.057	0.015 0.221*	0.097	0.022 0.429*
Asia	30 (75.0)	0.818	0.189 3.535	0.874	0.166 4.593
Latin America	7 (53.8)	0.318	0.059 1.705	0.43	0.060 3.094
Sub- Saharan Africa	53 (77.9)	0.964	0.238 3.905	2.883	0.532 15.637
Low incidence countries	11 (78.6)	1.0		1.0	

**Health check up**					
Yes	83 (43.0)	0.626	0.366 1.070	0.837	0.398 1.763
No	41 (54.7)	1.0		1.0	

**Have heard about TB before migration**					
Yes	81 (56.6)	2.491	1.518 4.089*	1.389	0.676 2.856
No	43 (34.4)	1.0		1.0	

**Received TB information in Sweden**					
Yes	47 (61.0)	2.319	1.349 3.987	0.726	0.314 1.676
No	77 (40.3)	1.0		1.0	

**Heard about the Communicable Disease Act**					
Yes	41 (66.1)	2.893	1.596 5.246	2.507	1.080 5.816*
No	83 (40.3)	1.0		1.0	1.0

**Knowledge level**					
High/good	30 (75.0)	4.277	1.995 9.169*	1.477	0.527 4.140
Low/bad	94 (41.2)			1.0	

*P < 0.05 (Univariate and multivariate logistic regression with attitude level as dependent variable)

### Perceived risk and seriousness of the disease, and information about TB in Sweden

Of the 268 respondents, 143(53%) reported having heard about TB before migration, but only 77 (29%) affirmed that they had ever received TB information in Sweden even though 193 (72%) reported having undergone medical screening. Those who claimed they received TB information after migration were asked how many times they got such information: 9 percent said they received it three times or more, 8 percent twice, and 11 percent once. The remaining 71 percent of the sample had never gotten information about TB since their arrival in Sweden. When asked about the source of that information, 17 percent mentioned schools, 11 percent health care services and just one respondent named a non-governmental organisation. If they had to seek TB information, 85 participants (32%) would turn to healthcare services and 3 (1%) somewhere else, but the remaining 67 percent did not know where to get information about TB. Similarly, knowledge about the Swedish Communicable Disease Act was very poor; only 23 percent of respondents reported having ever heard about this act. The majority (71%) requested information about TB in their mother tongues. International languages such as English, French or Spanish were options for not more than 17 percent.

The perceived risks of contracting or dying from TB while living in Sweden were quite low. Only 30 percent believed that a person living in Sweden was at some risk of contracting TB or dying from it if infected/diseased.

## Discussion

Our study of immigrant students indicates that despite exposure to the Swedish healthcare system during a routine screening process, basic knowledge related to TB is, in general, poor with several misconceptions and negative attitudes. These findings are similar to that of previous qualitative and quantitative studies conducted among high-risk populations, including migrant groups in different settings [15,21-25]. The most common misconceptions about TB among our respondents concerned transmission, contagiousness, differentiation of TB infection and disease, and prevention. As previously reported by other researchers, most of our respondents expressed an undue fear of catching and spreading the disease through social contacts and concerns about the consequences of others finding out in the event they contracted it [21-24,26-28]. In this study, respondents with tertiary, and those who had heard about TB before migration to Sweden, had better knowledge than their peers - this finding concurs with other studies that have found schooling an important determinant of TB knowledge [21,22,24,25]. One explanation is that literacy facilitates access to different sources of information including the internet and leaflets, and also makes it easier for those who access information to process and understand it. But, due to time constraints, it is a common practice of Swedish healthcare workers to provide clients/patients with health information in the form of leaflets in Swedish, or to advise them to seek information on the internet. Our results suggest that this practice may hinder those with low literacy and no computer skills to access health information [14,27,29]. Furthermore, the fact that we had to rely on interpreters to conduct this study, that 70 percent of respondents requested TB information in their mother tongues, and that they reported not knowing where to get information about TB, suggests that readily available information might currently be inaccessible to most respondents due to language barriers; this echoes other authors who have pointed out that language and literacy are substantial barriers to appropriate access and provision of healthcare services to immigrants [14,17,27,29,30]. Leaflets and internet are clearly useless for the less educated, and even for the literate the information needs to be provided in languages that can be clearly understood [17,27,29]. Migrants' educational background requires increased attention by healthcare providers when developing and providing TB information.

It is also evident from our results that previous exposure to TB information contributes to improved knowledge among respondents. Even if we could not satisfactorily control for the effect of TB history on knowledge, it is, however, an indication that TB education is likely to be successful in improving and sustaining knowledge [22,31]. Although improving knowledge does not necessarily lead to change in attitudes and behaviour, we agree with other researchers who stress that good knowledge is an essential component of looking after oneself and one's family and friends. However, the lack of effect of TB information and educational programmes could be ascribed to misconceptions about the disease, compounded with the fear of social rejection attached to having TB [15-17,26,31,32]. Ilongo emphasized the need of repeated educational interventions to influence this situation [31]. Nevertheless, the lack of significant difference in attitude between the highest and least educated groups could be attributed to the use of two types of data collection methods.

Several studies have also shown that fear of stigmatization in the community may also result in delays in seeking care, as well as poor treatment adherence [17,23,26-28,32]. In the current study, although the majority could correctly identify common symptoms of pulmonary TB, respondents also worried unjustifiably about contracting and spreading not only active disease, but also latent infection through physical contacts and sharing of objects. There was a great concern about others' reactions to a TB diagnosis. Ironically, because the perceived risk of contracting or dying from TB was low, this meant that those who could identify symptoms associated with TB may delay seeking healthcare, or that once the diagnosis is known, deny their illness to themselves or others, fearing stigmatization; they may also simply not perceive themselves at risk [17,21,24,26-28,31]. Yadav et al. described misconceptions and stigma associated with TB as: 'cultural barriers that provide a fertile ground for nurturing the persistence and spread of the disease' [25]. In their study, Kan et al. also stressed that TB stigma and cultural barriers might explain at least part of the poor outcome of case holding and contact investigation [7]. Such negative attitudes are detrimental, not only to the health of diseased people, but also to that of family members, close friends and the whole community [7,25-27,29]. These attitudinal problems need obviously to be addressed with repeated education.

Furthermore, as highlighted by Citrin [26], contact investigations amongst immigrant communities may be challenging if a diseased person or family with TB (that is sharing a dwelling with friends or other families) is unwilling to seek professional help for fear of rejection by their housemates and the wider community [7,26]. It would be difficult, if not impossible, to trace failed asylum seekers or undocumented migrants, who have no access to healthcare services and who fear being reported to the migration authorities [22]. One-fifth of our respondents reported undue fear of being removed from Sweden because of a TB diagnosis. Coker argued that immigration control and TB control are separate issues and should not be conflated because doing so would be counterproductive [33]. Negative attitudes observed among those who originated from the Middle East might, however, also express unfamiliarity with TB due to the relatively low prevalence of TB in this region compared to other regions such Sub-Saharan Africa and Asia [2].

Even though nearly three-quarters of our respondents had contact with Swedish healthcare professionals during the TB screening process, this did not result in knowledge improvement. Although the main purpose of this screening is to detect eligible cases for preventive and curative treatment, the process provides an opportunity for health workers to educate patients or clients and address those misconceptions revealed in this study [14]. Screening contributed only to 22 (4%) of the 554 cases reported in 2008, since most of the TB diagnoses (77%) were based on investigation of symptoms [10]. This could partly be explained by the fact that most active TB cases found in migrants seem to occur after resettlement [7,10-12,33]. Thus, offering TB information to those who come from high-burden countries would raise awareness about post-migration TB, reduce stigma and thereby allow early diagnosis and treatment of infectious cases to stop further transmission [17]. Our findings emphasize the need to include health education in the screening process; not only for those with active TB, but for all screened clients regardless of the Tuberculin skin test result - a position also recommended by the Swedish National Board of Health and Social Welfare [13].

Limitations of this study include the convenience sampling, the use of interpreters and the issues of validity and reliability of the TB knowledge questionnaire, which was primarily developed for the purpose of this study and thus might need further modification for use with other migrant groups. Respondents were selected conveniently and participation depended on parental consent (if < 18 years) and the availability of interpreters which particularly affect the response rate and may make the results difficult to generalize to other groups of migrants. The sample is, however, representative of newly-arrived immigrants from countries targeted with TB control interventions in Sweden. Furthermore, the self-reported nature of the questionnaire implies that we have to rely on respondents' honesty. The use of interpreters and two different methods for administering the questionnaire (researcher- and self-administered) may have affected the results, but these were the only ways to overcome language and educational barriers. Finally, though we collected information about arrival date, we were unable to use this information to assess the impact of duration of stay on knowledge and attitude, due to a large number of missing or inaccurate data. As TB is relatively unheard of in Sweden, it is quite possible that many people will be unaware of important knowledge that could help stop its increase. Future studies examining knowledge in relation to country of birth, history of TB, and exposure to TB education would give valuable information for the development of effective public health interventions.

## Conclusions

The extent of misconceptions and negative attitudes in our sample is a cause for concern, as it may negatively affect health-seeking behavior and compromise TB control in these communities. The results suggest that inadequate knowledge and negative attitudes towards TB persist even though the majority had had contact with health professionals through the screening process. This indicates that this opportunity is not being efficiently used to educate at-risk immigrants while readily-available information may currently be inaccessible to the majority of these immigrant groups due to language barriers and the usual disadvantages newcomers experience in terms of knowing where to access certain types of information. Appropriate and accessible educational programmes are needed to address misconceptions, to change attitudes and thus to improve TB control. Part of these findings and recommendations could also be relevant for other low-prevalence countries with high-incidence of TB among immigrants from high-burden countries, e.g. many industrialized countries in Western Europe, North America, and Australasia.

## Competing interests

The authors declare that they have no competing interests.

## Authors' contributions

FKKN participated in the design of the study, as well as the acquisition, documentation, analysis and interpretation of data, and the drafting of the article. CA and AKH participated in the design, coordination, and for important intellectual content. IK participated in the design of the study and the critical revision of the study. All authors read and approved the manuscript.

## Authors' information

FKKN: MD. MPH, Medical Doctor in Democratic Republic of Congo, Medical Interpreter in Sweden.

CA: MD. PhD, Associate professor, specialist in Infectious Diseases.

AKH: MD. DrPH, Associate professor in Public Health.

IK: MD, DTMH, professor em, specialist in Infectious Diseases, PhD Medical Ethics.

## Pre-publication history

The pre-publication history for this paper can be accessed here:

http://www.biomedcentral.com/1471-2458/10/349/prepub
